# Alzheimer’s polygenic risk scores, *APOE*, Alzheimer’s disease risk, and dementia-related blood biomarker levels in a population-based cohort study followed over 17 years

**DOI:** 10.1186/s13195-023-01277-8

**Published:** 2023-07-29

**Authors:** Hannah Stocker, Kira Trares, Léon Beyer, Laura Perna, Dan Rujescu, Bernd Holleczek, Konrad Beyreuther, Klaus Gerwert, Ben Schöttker, Hermann Brenner

**Affiliations:** 1 Network Aging Research, Heidelberg University, Heidelberg, Germany; 2 Division of Clinical Epidemiology and Aging Research, German Cancer Research Center, Heidelberg, Germany; 3 Center for Protein Diagnostics (ProDi), Ruhr-University Bochum, Bochum, Germany; 4 Department of Biophysics, Ruhr-University Bochum, Bochum, Germany; 5 Department of Translational Research in Psychiatry, Max Planck Institute of Psychiatry, Munich, Germany; 6 Department of Psychiatry, Medical University of Vienna, Vienna, Austria; 7 Saarland Cancer Registry, Saarbrücken, Germany

**Keywords:** Alzheimer’s disease, Polygenic risk scores, Blood-biomarkers

## Abstract

**Background:**

In order to utilize polygenic risk scores (PRSs) for Alzheimer’s disease (AD) in a meaningful way, influential factors (i.e. training set) and prediction across groups such as *APOE e4 (APOE4)* genotype as well as associations to dementia-related biomarkers should be explored. Therefore, we examined the association of *APOE4* and various PRSs, based on training sets that utilized differing AD definitions, with incident AD and all-cause dementia (ACD) within 17 years, and with levels of phosphorylated tau181 (P-tau181), neurofilament light (NfL), and glial fibrillary acidic protein (GFAP) in blood. Secondarily, effect modification by *APOE4* status and sex was examined.

**Methods:**

In this prospective, population-based cohort study and nested case–control study, 9,940 participants in Germany were enrolled between 2000 and 2002 by their general practitioners and followed for up to 17 years. Participants were included in this study if dementia status and genetic data were available. A subsample of participants additionally had measurements of P-tau181, NfL, and GFAP obtained from blood samples. Cox and logistic regression analyses were used to assess the association of genetic risk (*APOE* genotype and PRS_noAPOE_) with incident ACD/AD and log-transformed blood levels of P-tau181, NfL, and GFAP.

**Results:**

Five thousand seven hundred sixty-five participants (54% female, aged 50-75years at baseline) were included in this study, of whom 464 received an all-cause dementia diagnosis within 17 years. The PRSs were not more predictive of dementia than *APOE4*. An *APOE4* specific relationship was apparent with PRSs only exhibiting associations to dementia among *APOE4* carriers. In the nested case–control study including biomarkers (*n* = 712), *APOE4* status and polygenic risk were significantly associated to levels of GFAP in blood.

**Conclusions:**

The use of PRSs may be beneficial for increased precision in risk estimates among *APOE4* carriers. While *APOE4* may play a crucial etiological role in initial disease processes such as Aβ deposition, the PRS may be an indicator of further disease drivers as well as astrocyte activation. Further research is necessary to confirm these findings, especially the association to GFAP.

**Supplementary Information:**

The online version contains supplementary material available at 10.1186/s13195-023-01277-8.

## Introduction

Genetic predisposition plays a fundamental role in the development of Alzheimer’s disease (AD) with heritability of late-onset AD estimated as high as 79% [1–3]. The greatest known genetic risk factor for AD is the e4 allele of Apolipoprotein E (*APOE4),*with heterozygotes and homozygotes experiencing a three and 15-fold increased risk of AD development, respectively [2]. In recent years, large genome-wide association studies (GWAS) for AD have provided more information regarding the genetic landscape of AD with many genetic loci contributing to AD risk, albeit with much smaller effects than *APOE4* [4–6].

Polygenic risk scores (PRSs) have been used to summarize collective genetic risk and can discriminate AD, but may not be more predictive than *APOE4 *alone [3, 7–9]. The most recent research has however shown increased diagnosis prediction accuracy [5, 10–13]. PRSs have shown mixed associations to AD-related biomarkers [14–20]. *APOE* has been consistently associated to Aβ measured in cerebrospinal fluid or by positron emission tomography (PET) imaging [15, 21, 22], while PRSs excluding *APOE* often exhibited a lack of association to Aβ [18, 19, 21]. Very limited research regarding the association to blood biomarkers exists [17].

In order to apply PRSs meaningfully, it is critical to investigate factors that may influence risk estimates including the training set of the PRS, specifically the definition of AD used in the training set. In addition to the consideration of training set, the use of PRSs in specific subgroups based upon factors such as *APOE4* status or sex, may provide more precise risk estimates. For example, it has been shown that the age of symptom onset was differentiated by PRS only in *APOE4 *carriers [23]. Furthermore, there is evidence that *APOE4* and AD PRSs have sex-specific effects influencing both overall risk and age of symptom development [24].

Therefore, the aim of this study was to investigate the association of *APOE4* and two PRSs based upon training sets, which utilize differing AD definitions, with incident AD and all-cause dementia (ACD) diagnosis within 17 years as well as with levels of the AD-related blood biomarkers, phosphorylated tau181 (P-tau181), neurofilament light (NfL), and glial fibrillary acidic protein (GFAP) at baseline before dementia diagnosis in a community-based cohort study. Secondarily, effect modification by *APOE4* status and sex was investigated.

## Methods

### Study participants and data collection

The ESTHER study (German name: *Epidemiologische Studie zu Chancen der Verhütung, Früherkennung und optimierten Therapie chronischer Er-krankungen in der älteren Bevölkerung*) is a population-based prospective cohort study of community-dwelling older adults in Germany. Briefly, ESTHER consists of 9,940 participants (50–75 years old at baseline) recruited by general practitioners (GPs) in a statewide study in Saarland, a small state (approximately 1 million inhabitants) located in southwest Germany, in 2000–2002 [8, 25]. The ESTHER study was approved by the Ethics Committee of the Medical Faculty at Heidelberg University and the Physicians’ Board of Saarland in accordance with the declaration of Helsinki. Written informed consent was obtained from all participants.

Dementia diagnoses were collected from participants’ GPs during the 14 and 17-year follow-ups as previously described [8, 26]. More details regarding the ESTHER study and the dementia diagnoses can be found in Supplementary Text 1.

The sample for this study included *n* = 5,765 ESTHER participants with available dementia and genetic information (Supplementary Fig. 1). The analyses with the AD related blood biomarkers, P-tau181, GFAP, and NfL, were conducted within a previously defined nested case–control study (*n* = 768) in ESTHER [27] that included all AD cases until the 17-year follow-up and several vascular dementia and mixed dementia cases for comparison. Not all dementia cases were measured due to limited resources. In this analysis, study participants without available genetic information (*n* = 53) or without usable GFAP or NfL measurements (*n* = 3) were excluded (Supplementary Fig. 1).

### Laboratory measurements and imputation

Genotyping, P-tau181, GFAP, and NfL measurements were carried out as previously described in blood samples that were taken during a routine health examination at baseline and stored at − 80 °C until analysis [8, 27]. Additionally, serum creatinine and cystatin C measurements were completed and kidney function was assessed through the estimated glomerular filtration rate (eGFR), estimated by the 2021 Chronic Kidney Disease Epidemiology Collaboration creatinine-cystatin C (eGFRcr-cys) Eq [28]. Details regarding all laboratory measurements and genetic imputation can be found in Supplementary Text 2.

### Polygenic risk score calculation

The two PRSs in this study were weighted scores including AD associated single-nucleotide polymorphisms (SNPs) reaching genome-wide significance in the Kunkle et al. and Bellenguez et al. GWAS meta-analyses. The scores were calculated by summing the number of risk alleles weighted by the magnitude of association (ln of the odds ratio (OR)) from Kunkle et al. and Bellenguez et al. [5, 6] SNPs reaching genome-wide significance in the summary statistics of each GWAS (Kunkle et al. & Bellenguez et al.) were extracted from the imputed ESTHER data and the following quality control steps were carried out: 1) participant genotype missing threshold of 10%; 2) minor allele frequency threshold of 0.01, 3) SNP missing threshold of 5%, and 4) Hardy–Weinberg equilibrium threshold of 10^–6^. Linkage disequilibrium-based clumping was then carried out, providing the most significantly associated SNP in each region of linkage disequilibrium (using PLINK clumping command with a pairwise r^2^ threshold of 0.2). Then, SNPs within or directly upstream/downstream from the *APOE* locus (chr19: 45,404,000–45,418,000) were excluded and those reaching genome-wide significance were utilized in each PRS, resulting in 55 SNPs (Kunkle PRS) and 105 SNPs (Bellenguez PRS). The median imputation quality (R^2^) for the included SNPs was 0.92 and 0.95 for the Kunkle PRS and Bellenguez PRS, respectively. The SNP extraction, quality control, and PRS calculation were completed using PLINK 1.9 (https://pngu.mgh.harvard.edu/purcell/plink/) [29]. The PRSs were calculated in PLINK using the –score function. By default, missing genotypes contribute an amount proportional to the loaded or imputed allele frequency. A list of included SNPs can be found in Supplementary Table 1.

### Statistical analysis

Baseline characteristics of participants were summarized using descriptive statistics. The only covariate with missing data was *APOE* genotype (0.6% missing) and in analyses utilizing *APOE*, participants with missing information were excluded. Multivariate logistic and linear regression analyses were used to calculate ORs and 95% confidence intervals (CIs) or beta coefficients and p-values to investigate the association of the genetic risk predictors (*APOE*, Kunkle PRS, Bellenguez PRS) with: 1) incident AD and ACD diagnosis within 17 years; and 2) the blood biomarkers, P-tau181, NfL, and GFAP. *APOE* status was utilized as a binary variable (*APOE4* + : ≥ 1 ε4 allele vs. *APOE*-: no ε4 allele), while each PRS was considered per SD increase in score and as quartiles, calculated using the entire sample. Both PRSs were normally distributed (Supplementary Fig. 2). T-tests and ANOVA were used to detect significant differences in age at diagnosis based upon *APOE4* (binary) and each PRS (quartiles). The PRSs did not include any SNPs in or around the *APOE* locus in order to compare the predictive value of *APOE e4* alone to the PRSs.

Covariates for all logistic and linear regression analyses included age, sex, and ten principal components. The analyses utilizing the blood biomarkers, P-tau181, NfL, and GFAP, were additionally adjusted for eGFRcr-cys [28] and the blood biomarkers were considered continuously and as binary outcomes, comparing the highest quintile of levels to the lower four quintiles (Q5vsQ1-4). Mann–Whitney U tests were used to compare biomarker levels. Correlation between log-transformed biomarker levels and each PRS was assessed with Pearson correlation coefficients.

Receiver operating characteristic curves (ROC) and resulting c-statistics were calculated for AD and ACD diagnosis within 17 years based upon: 1) age and sex; 2) age, sex and *APOE*; 3) age, sex, and Kunkle PRS; and 4) age, sex, and Bellenguez PRS. Additionally, c-statistics for age, sex, *APOE*, and each PRS were calculated to determine if either PRS improved disease prediction accuracy beyond *APOE*. ROC contrast analysis using the DeLong test was conducted to compare for significant differences between curves [30].

Additionally, stratified and interaction analyses by *APOE4* status and sex were completed for all outcomes. All analyses were conducted using SAS software, version 9.4 (SAS Institute, 128 Cary, NC). Statistical tests were two sided and conducted at an α-level of 0.05.

## Results

### Participant characteristics

A total of 5,765 participants from the ESTHER study had available dementia and genetic information, of whom 464 received an ACD diagnosis and 153 an AD diagnosis within 17 years, while 5301 participants remained without dementia diagnosis throughout follow-up (Supplementary Fig. 1). The mean length of follow-up was 10.9 in incident dementia cases and 15.1 years in participants that remained without dementia diagnosis. AD and ACD diagnoses occurred on average 10.4 and 10.9 years after baseline, respectively. The mean age of participants at baseline was 64 years (age range 50–75 years) and there were slightly more females (53%) than males (Table 1). Half of participants with AD diagnosis, 39% of ACD participants, and 25% of participants without dementia diagnosis throughout follow-up had one or more *APOE* e4 alleles. More participants with AD and ACD diagnosis were in the highest quartile of both the Kunkle PRS and the Bellenguez PRS than participants without dementia.

**Table 1:** Participant characteristics and association to incident Alzheimer’s disease and all-cause dementia diagnosis within 17 years

Predictor	**Alzheimer’s disease** **n (%)**	**All-cause dementia** **n (%)**	**Participants without dementia diagnosis** **n (%)**	**Alzheimer’s disease** **OR (95% CI) *p*-value**	**All-cause dementia** **OR (95% CI) *p*** **-alue**
n	153	464	5301	-	-
Age, mean ± SD	66.6 ± 5.2	66.9 ± 5.2	61.3 ± 6.4	**1.16 (1.12–1.24)** < .0001	**1.17 (1.15–1.19)** < .0001
Male	67 (43.8)	227 (48.9)	2423 (45.7)	Ref	Ref
Female	86 (56.2)	237 (51.1)	2878 (54.3)	1.12 (0.81–1.55) .55	0.89 (0.73–1.09) .26
*APOE4* –	76 (50)	280 (60.7)	3970 (75.4)	Ref	Ref
*APOE4* +	76 (50)	181 (39.3)	1299 (24.7)	**3.27 (2.35–4.55)** < .0001	**2.14 (1.74–2.63)** < .0001
Kunkle PRS					
Q1	27 (17.7)	103 (22.2)	1338 (25.2)	Ref	Ref
Q2	32(20.9)	102 (22.0)	1339 (25.3)	1.10 (0.65–1.86) .71	0.92 (0.69–1.24) .59
Q3	35 (22.9)	103 (22.2)	1339 (25.3)	1.20 (0.72–2.00) .49	0.91 (0.68–1.21) .51
Q4	59 (38.6)	156 (33.6)	1285 (24.2)	**2.19 (1.37–3.51) .001**	**1.49 (1.14–1.96) .004**
Bellenguez PRS					
Q1	24 (15.7)	98 (21.1)	1343 (25.3)	Ref	Ref
Q2	39 (25.5)	105 (22.6)	1336 (25.2)	1.63 (0.97–2.75) .06	1.09 (0.81–1.46) .58
Q3	40 (26.1)	122 (26.3)	1320 (24.9)	1.68 (1.001–2.83)** .048**	1.23 (0.92–1.64) .15
Q4	50 (32.7)	139 (30.0)	1302 (24.6)	**2.09 (1.27–3.44) .0039**	**1.38 (1.04–1.82) .03**
Kunkle PRS per SD increase	-	-	-	**1.51 (1.30–1.75)** < .0001	**1.30 (1.12–1.36)** < .0001
Bellenguez PRS per SD increase	-	-	-	**1.32 (1.11–1.56) .001**	**1.14 (1.03–1.26) .01**

*Note*: Logistic regression analyses adjusted for age, sex, and 10 principal components. Bold values denote statistical significance at the *p* < .05 level

*Abbreviations*: *APOE* e4 + 1 or more e4 alleles, *APOE* e4—no e4 alleles, *CI* confidence interval, *OR* odds ratio, *SD* standard deviation, *Q* quartile

The AD-related blood biomarker sample included 712 participants without dementia diagnosis at baseline, of whom 239 participants received a dementia diagnosis and 470 remained without dementia diagnosis throughout 17 years of follow-up (Supplementary Fig. 1). The blood biomarker measurements were completed in blood drawn at baseline. Participants with baseline blood biomarker levels in the highest quintile were more often *APOE4* carriers than non-carriers, P-tau181 (40% vs. 29%), NfL (34% vs. 31%), and GFAP (44% vs. 29%) (Supplementary Table 2).

### Alzheimer’s disease, all-cause dementia, and the genetic risk predictors

*APOE4* carriers had higher odds of incident AD and dementia diagnosis within 17 years compared to non-carriers (Table 1, OR, 95% CI: AD, 3.27, 2.35–4.55; ACD: 2.14, 1.74–2.63). Participants within the highest quartile of the Kunkle and Bellenguez PRS also experienced increased odds of AD and ACD diagnosis (OR, 95% CI: Kunkle PRS AD, 2.19, 1.37–3.51; Bellenguez PRS AD: 2.09, 1.27–3.44; Kunkle PRS ACD: 1.49, 1.14–1.96; Bellenguez PRS ACD: 1.38, 1.04–1.82). Participants in lower genetic risk categories (APOE4-, PRSQ1) were older at diagnosis of AD and ACD than participants in higher genetic risk categories (*APOE4* + , PRSQ4), although a statistically significant difference in age at diagnosis was only evident according Kunkle PRS quartiles and only for AD diagnosis (Supplementary Table 3).

**Fig. 1:**
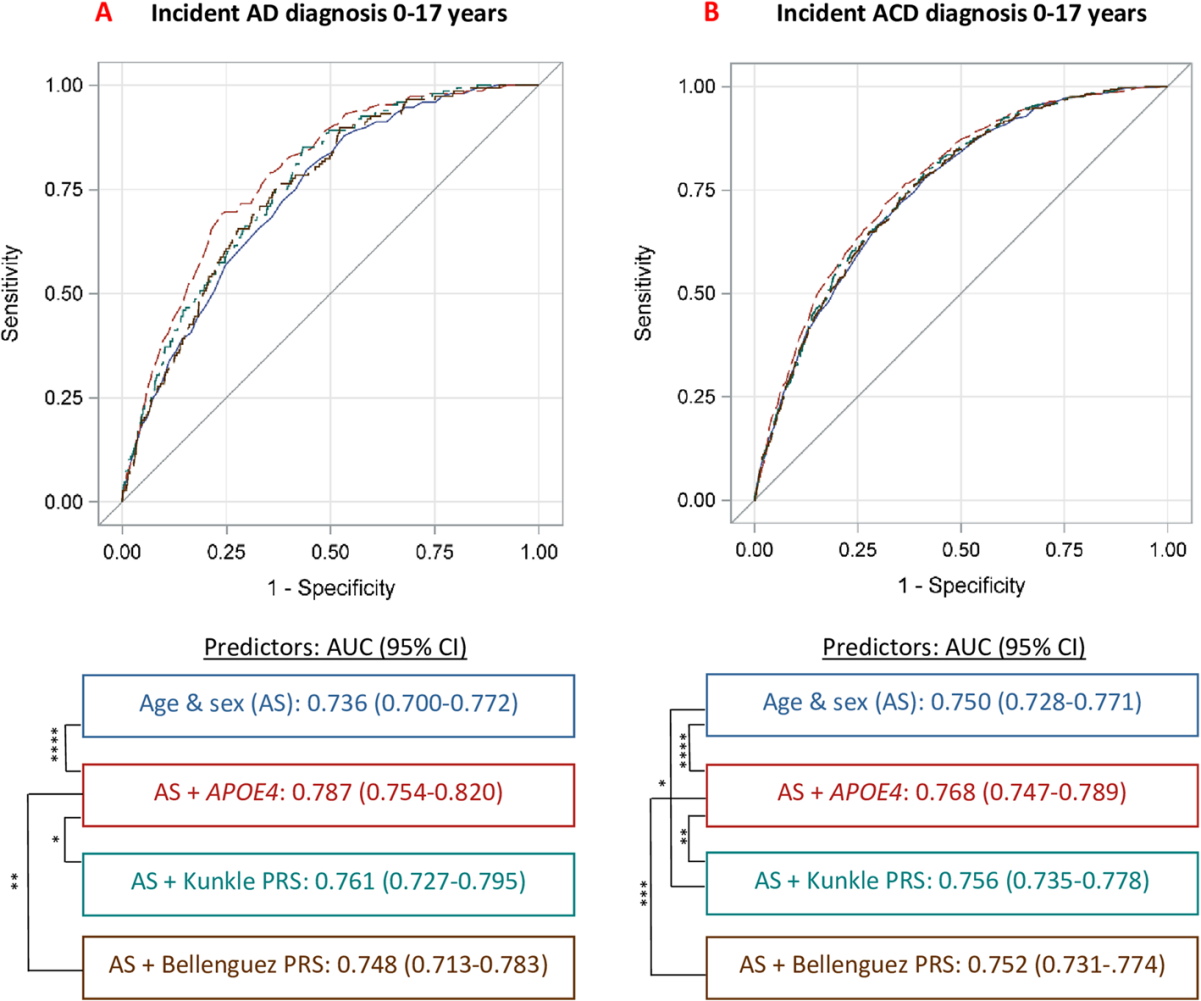
ROC curves and contrast for incident Alzheimer’s disease (AD) and all-cause dementia (ACD) diagnosis within 17 years based upon age, sex, and the genetic risk predictors. Area under the ROC curve (AUC) including 95% confidence intervals (CIs) are reported below ROC curves. ROC contrast analysis using the DeLong test was conducted to compare for significant differences between curves as indicated by: *p < .05, **p < .01, ***p < .001, ****p < .0001

The greatest disease prediction accuracy of AD diagnosis was achieved by age, sex, and *APOE4* status (c-statistic, 95% 95% CI: 0.787, 0.754–0.820), which was significantly greater than prediction by age, sex, and either PRS (Fig. 1). A similar pattern was evident for ACD with *APOE4* as the most accurate predictor followed by the Kunkle PRS. The addition of either PRS to a model including age, sex, and *APOE* did not improve AD or ACD prediction accuracy (c-statistic, 95%CI: AD: age + sex + APOE + Kunkle PRS: 0.787, 0.754–0.820; age + sex + *APOE* + Bellenguez PRS: 0.788, 0.755–0.821; ACD: age + sex + *APOE* + Kunkle PRS: 0.768, 0.747–0.789; age + sex + *APOE* + Bellenguez PRS: 0.768, 0.747–0.789).

### Blood biomarkers, P-tau181, NfL, and GFAP, and the genetic risk predictors

**Fig. 2:**
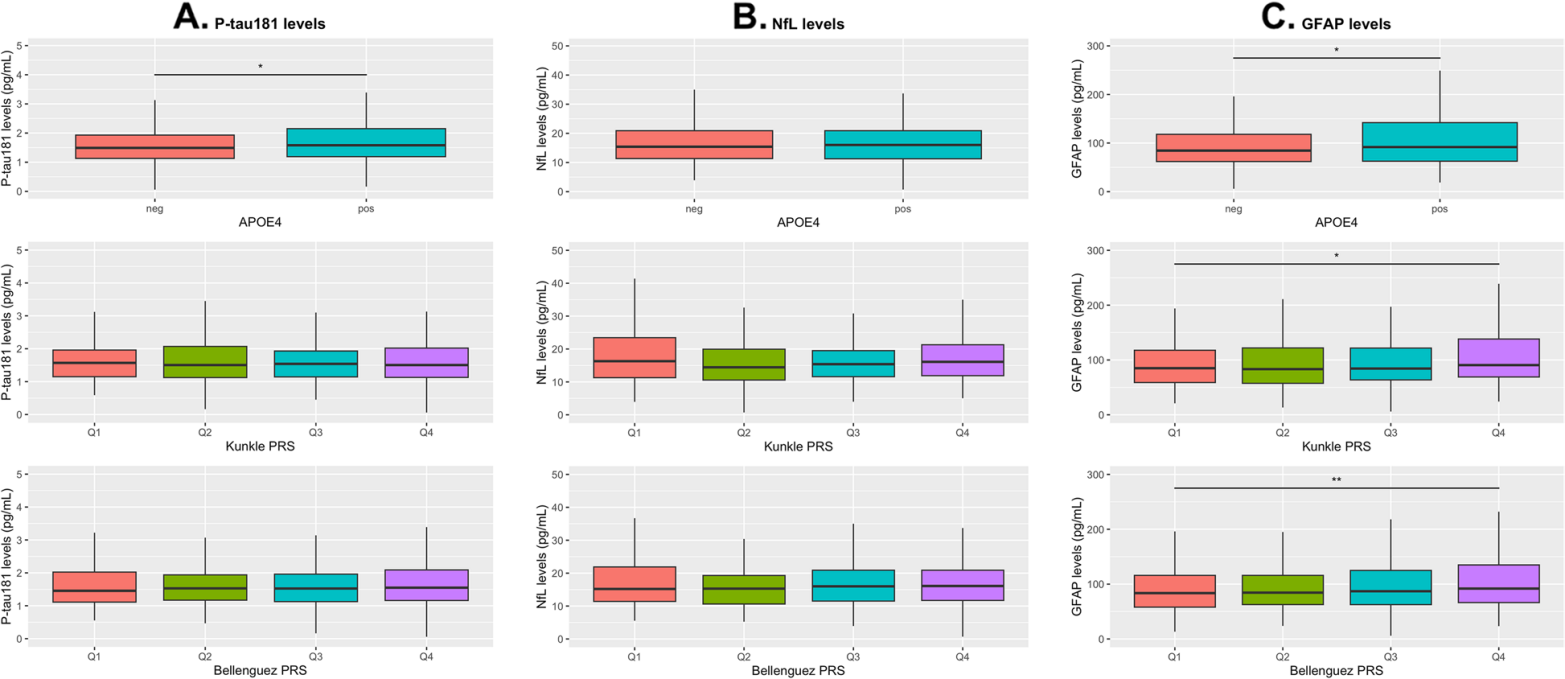
Baseline blood biomarker levels at baseline (before dementia diagnosis) according to *APOE4* or PRS quartile. **A**, Baseline P-tau181 levels; **B**, Baseline NfL levels; **C**, Baseline GFAP levels. Q, quartile. Mann–Whitney U tests were used to test for statistically significant differences by APOE4 status or PRS quartile as indicated by: *p < .05; **p < .01

P-tau181 and GFAP levels at baseline were significantly higher in *APOE4* carriers than non-carriers. The only blood biomarker to have significantly different levels by PRS quartile was GFAP (Fig. 2). In linear regression analyses, only GFAP levels were significantly associated to *APOE4* status and the Kunkle PRS (beta, p-value: *APOE4*, 0.07, 0.049; Kunkle PRS, 0.05, 0.008) (Table 2). In the logistic regression analyses, participants that were *APOE4* carriers had higher odds of having P-tau181 and GFAP levels in the top quintile (Supplementary Table 2, OR, 95% CI: P-tau181, 1.60, 1.07–2.39; GFAP, 1.95, 1.28–2.97) but not NfL levels in the top quintile (OR, 95% CI: 1.14, 0.72–1.81). The Kunkle PRS was significantly associated to GFAP levels (per SD increase, OR, 95% CI: 1.23, 1.01–1.48), but not P-tau181 or NfL levels. GFAP levels were significantly correlated to both PRSs, albeit weakly (Supplementary Fig. 3).

**Table 2:** Linear regression results: Association of *APOE4,* Kunkle PRS, and Bellenguez PRS with blood-based biomarkers, P-tau181, NfL, & GFAP

Predictor	**n**	***P-tau181*** **beta (*p*-value)**	***NfL*** **beta (*p*-value)**	***GFAP*** **beta (*p*-value)**
n total	712			
***APOE4* –**	486	Ref	Ref	Ref
***APOE4* + **	224	0.06 (.11)	-0.04 (.15)	**0.07 (.049)**
Kunkle PRS				
Q1	156	Ref	Ref	Ref
Q2	188	0.03 (.58)	-0.07 (.07)	0.03 (.58)
Q3	167	-0.01 (.81)	-0.08 (.06)	0.009 (.87)
Q4	201	-0.01 (.83)	-0.06 (.12)	0.08 (.11)
Bellenguez PRS				
Q1	148	Ref	Ref	Ref
Q2	184	0.03 (.54)	-0.08 (.06)	0.04 (.42)
Q3	187	-0.02 (.72)	-0.08 (.07)	0.05 (.35)
Q4	193	-0.008 (.88)	-0.03 (.41)	0.12 (0.20)
Kunkle PRS per SD increase	712	0.004 (.82)	0.01 (.43)	**.05 (.008)**
Bellenguez PRS per SD increase	712	0.005 (.78)	-0.02 (.11)	0.03 (.07)

*Note*: Linear regression analyses adjusted for age, sex, 10 principal components, and estimated glomerular filtration rate according to the 2021CKD-EPI creatinine-cystatin C equation (eGFRcr-cys). Bold values denote statistical significance at the *p* < .05 level

*Abbreviations*: *APOE4* + 1 or more e4 alleles, *APOE*4—no e4 alleles, *Q* quartile

### *APOE4* and sex stratified analyses

Significant interaction between both PRSs and *APOE4* status was evident (Table 3). Increased PRS values were associated with increased odds of AD and ACD diagnosis only among *APOE4* carriers. Among *APOE4* carriers both PRSs performed similarly (Table 3). Participants experienced 1.45- and 1.49-times greater odds of incident AD diagnosis per SD increase in Kunkle and Bellenguez PRS, respectively (OR, 95% CI: Kunkle PRS, 1.45, 1.17–1.79; Bellenguez PRS AD: 1.49, 1.16–1.92). There was no significant interaction between the genetic predictors and sex in the association to AD or ACD (Supplementary Table 4).

**Table 3:** Association of polygenic risk scores with Alzheimer’s disease, all-cause dementia, and blood biomarkers stratified by *APOE* status

	*APOE4* +			*APOE4-*			
	Total n	Cases n	OR (95% CI) *p*-value	Total n	Cases n	OR (95% CI) *p*-value	Interaction *p*-value
*Alzheimer’s disease*							
Kunkle PRS per SD increase	1375	76	**1.45 (1.17–1.79) .0006**	4046	76	0.72 (0.49–1.07) .12	.001
Bellenguez PRS per SD increase	1375	76	**1.49 (1.16–1.92) .002**	4046	76	0.94 (0.75–1.19) .62	< .01
*All-cause dementia*							
Kunkle PRS per SD increase	1480	181	**1.22 (1.06–1.42) .008**	4250	280	**0.84 (0.72–0.99) .04**	< .001
Bellenguez PRS per SD increase	1480	181	**1.35 (1.14–1.61) .0007**	4250	280	0.93 (0.82–1.06) .27	< .001
*P-tau181*			*beta (p-value)*			*beta (p-value)*	
Kunkle PRS per SD increase	224		-0.02 (.52)	486		-0.005 (.88)	.71
Bellenguez PRS per SD increase	224		0.004 (.91)	486		-0.01 (.60)	.40
*NfL*							
Kunkle PRS per SD increase	224		-0.002 (.95)	483		-0.03 (.16)	.14
Bellenguez PRS per SD increase	224		0.01 (.65)	483		-0.02 (.28)	.08
*GFAP*							
Kunkle PRS per SD increase	224		0.02 (.44)	483		0.02 (.57)	.42
Bellenguez PRS per SD increase	224		0.05 (.19)	483		0.03 (.13)	.12

*Note*: Logistic and linear regression analyses adjusted for age, sex, and 10 principal components. The analyses with the blood biomarkers as outcomes additionally adjusted for the estimated glomerular filtration rate according to the 2021CKD-EPI creatinine-cystatin C equation (eGFRcr-cys). Bold values denote statistical significance at the *p* < .05 level

*Abbreviations*: *APOE*4 + 1 or more e4 alleles, *APOE*4—no e4 alleles, *CI* confidence interval, *OR* odds ratio, *SD* standard deviation

In the subsample including AD-related blood biomarkers, there was no significant interaction between *APOE4* and the PRSs (Table 3). The sex stratified analyses revealed that GFAP levels were only statistically significantly associated to levels among men (Supplementary Table 4).

## Discussion

Two PRSs based upon different GWASs with discrepant definition of AD were significantly associated with incident AD and ACD diagnoses, but did not exhibit greater disease prediction accuracy than *APOE4* status alone in a community-based study followed over 17 years. An *APOE4* specific relationship was apparent with significant associations between PRS and AD diagnosis evident only among *APOE4* carriers. *APOE4* status was associated to P-tau181 and GFAP, but not NfL levels in blood at baseline (0–17 years before dementia diagnosis). Finally, the Kunkle PRS was also significantly associated to GFAP levels in blood at baseline and this relationship was modified by sex, with significant associations evident only among males.

### Alzheimer’s disease, all-cause dementia, and the genetic risk predictors

Over the last decade, many different PRSs for AD have been developed and validated [7, 8, 10, 12, 23]. Still, *APOE4 *remains the greatest predictor of AD diagnosis, with little added discrimination ability of PRSs [7, 10]. PRSs may however be particularly useful in determining individuals at risk among *APOE4* carriers. In our study, in analyses stratified by *APOE4* status, we found the PRS was only significantly associated to AD and ACD diagnosis among *APOE4* carriers, which is in line with previous research [23]. Not all *APOE4* carriers develop AD and PRSs may provide insight into which *APOE4* carriers are more likely to develop symptoms.

Another important factor in PRS analyses includes the choice of training dataset. We found that the Kunkle PRS predicted AD diagnosis more accurately than the Bellenguez PRS in the ESTHER cohort, which could be due to the definition of AD as clinical diagnosis in the Kunkle GWAS, while the Bellenguez GWAS also included AD by proxy cases [5, 6]. Intriguingly, the PRSs exhibited similar and significant associations to AD diagnosis among *APOE4* carriers. Although the Bellenguez PRS included almost double the SNPs of the Kunkle PRS, it performed similarly. The SNPs in the PRS (excluding *APOE*) have small effect sizes and it has been shown that the variance explained by SNPs other than *APOE* may be less than 2% [31], possibly explaining the relatively small difference in disease prediction by the two PRSs.

The age at onset of AD has also been shown to differ based upon genetic makeup [23, 31]. Although it is known that diagnoses are likely delayed in the community, in our study there was still a difference in age at diagnosis by genetic risk category, suggesting the possible utility of genetic risk predictors in models that predict age at onset of clinical symptoms.

### Blood biomarkers, P-tau181, NfL, and GFAP, and the genetic risk predictors

Previous studies have exhibited mixed results regarding the association between PRSs and AD biomarkers. Studies with PRSs including *APOE* showed positive associations to Aβ deposition [15, 32–34]; while those PRSs excluding *APOE* were less consistent often lacking significant associations to Aβ [14, 18, 19, 21, 35]. It has been theorized that *APOE *may be at least in part responsible for amyloid accumulation, while the genetic loci included in the PRSs may influence other drivers of disease progression [16, 21]. Previous evidence regarding the association between PRS and blood biomarker levels is limited [17, 36]. One study showed a positive association between PRS excluding *APOE *and P-tau181 levels only among participants with mild cognitive impairment [17]. The associations between PRS and NfL/GFAP levels in blood in a population of European descent have not been previously investigated.

In our study, participants had higher odds of P-tau181 levels in the highest quintile if *APOE4* positive. P-tau181 rises in response to amyloid deposition, further supporting the previous associations between *APOE* and amyloid. Both *APOE4* and the Kunkle PRS that did not include *APOE* were associated to baseline GFAP levels, a marker of astrocyte activation, indicating that both genetic risk predictors may have a role in astrocyte activation and GFAP may be in part a marker of the heritable component of disease etiology.

There was no interaction in our study between *APOE* and the PRS in the association to the AD related biomarkers as was seen in the association to AD diagnosis. Interestingly, we saw evidence of effect modification by sex in the association between the genetic risk predictors and GFAP levels, with associations only evident among males. GFAP levels have been shown to differ according to sex [37] and astrocytic response may be affected by sex hormones [38], possibly explaining differences in GFAP levels.

### Implications

This study further confirms previous work that AD PRSs are not more predictive of AD than *APOE*, however may add additional information regarding AD risk among *APOE4* carriers, the age at which symptoms begin to occur, and possibly regarding astrocyte activation. While *APOE4* and the PRSs were both associated to clinical AD diagnosis, only *APOE4* was associated to P-tau181 levels in blood years before diagnosis, further supporting the theory that *APOE4* has a crucial etiological role in amyloid deposition and other genetic risk loci may support further pathological processes in disease progression. Both *APOE4* and the PRS were associated to GFAP levels and while astrocyte activation may occur in response to amyloid accumulation, explaining the association to *APOE4*, it may also occur due to additional genetic predisposition. PRSs may be useful in the research setting for more specialized risk stratification among *APOE4* carriers. Further work is necessary to determine clinical applications of PRSs in the future, whether the information added by the AD PRSs would be useful in determining treatment.

### Strengths and weaknesses

The strengths of this study include the novel investigations and findings regarding the comparison of PRSs based upon the most recent and largest GWAS as well as the association to NfL and GFAP blood levels, which has not been previously explored in a population of European descent. Additionally, the large population-based sample set in the community, which may be more representative than specialized studies, with extensive follow-up adds a unique and important perspective to the AD genetic risk and biomarker literature.

Limitations of this study include the possibility of dementia misdiagnosis/underdiagnosis or delayed diagnosis, a lack of statistical power among stratified analyses especially within the nested case–control study, a limited amount of biomarker measurements, and limited generalizability of the study to individuals of European descent. The dementia diagnoses in the ESTHER study were clinical diagnoses reported heterogeneously by numerous practitioners, which is the nature of community-based cohort studies that portray common practice in such a setting. The strength of the diagnoses in the ESTHER study are however supported by the *APOE*ε4/AD PRS distribution among dementia diagnoses that closely mirror established literature [8].

## Conclusion

In this large community-based study, two PRSs based upon different GWAS did not add to AD predictive ability above and beyond *APOE*, however, may add important information regarding AD risk among *APOE4* carriers. Furthermore, *APOE4* status was associated to P-tau181 and GFAP levels at baseline, while the PRS was also associated to GFAP levels. The use of PRSs may be beneficial for increased precision in risk estimates, especially among *APOE4* carriers, and GFAP may be an important early predisposition marker of AD. Further research should confirm these results especially the association to GFAP levels.

## Supplementary Information

**Additional file 1: Supplementary Text 1. Supplementary Text 2. Supplementary Figure 1.** Flow chart of ESTHER participants included in the study. **Supplementary Figure 2.** Distribution of the polygenic risk scores (Kunkle et al. 2019 and Bellenguez et al. 2022) in the ESTHER study. **Supplementary Table 1.** SNPs included in the polygenic risk scores. **Supplementary Figure 3.** Correlation between the polygenic risk scores and AD related blood biomarkers. **Supplementary Table 2.** Participant characteristics and association to blood-based biomarkers, P-tau181, NfL, & GFAP. **Supplementary Table 3.** Age at diagnosis according to APOE4 status and PRS quartile. **Supplementary Table 4.** Association of APOE and PRSs with Alzheimer’s disease, All-cause dementia, and blood biomarkers stratified by sex.

## Data Availability

The datasets generated and/or analyzed during the current study are not publicly available due to local regulations but may be made available from the ESTHER study principal investigator upon reasonable request.

## Abbreviations

ACD: All-cause dementia
AD: Alzheimer’s disease
APOE4: APOE e4 allele
CI: Confidence interval
GFAP: Glial fibrillary acidic protein
GWAS: Genome-wide association study
GP: General practitioner
eGFR: Estimated glomerular filtration rate
eGFRcr-cys: Estimated glomerular filtration rate based upon creatinine and cystatin C
NfL: Neurofilament light
OR: Odds ratio
PRS: Polygenic risk score
P-tau181: Phosphorylated tau181
SNP: Single-nucleotide polymorphism
