# Supplementary material for: Alzheimer’s polygenic risk scores, APOE, Alzheimer’s disease risk, and dementia-related blood biomarker levels in a population-based cohort study followed over 17 years
Source: Alzheimers Res Ther. 2023 Jul 29;15:129. doi: 10.1186/s13195-023-01277-8 (PMC10386275; doi:10.1186/s13195-023-01277-8)
Supplement: Supplementary file 1 — Additional file 1: Supplementary Text 1. Supplementary Text 2. Supplementary Figure 1. Flow chart of ESTHER participants included in the study. Supplementary Figure 2. Distribution of the polygenic risk scores (Kunkle et al. 2019 and Bellenguez et al. 2022) in the ESTHER study. Supplementary Table 1. SNPs included in the polygenic risk scores. Supplementary Figure 3. Correlation between the polygenic risk scores and AD related blood biomarkers. Supplementary Table 2. Participant characteristics and association to blood-based biomarkers, P-tau181, NfL, & GFAP. Supplementary Table 3. Age at diagnosis according to APOE4 status and PRS quartile. Supplementary Table 4. Association of APOE and PRSs with Alzheimer’s disease, All-cause dementia, and blood biomarkers stratified by sex. [file 13195_2023_1277_MOESM1_ESM.docx]

Alzheimer’s polygenic risk scores, *APOE*, Alzheimer’s disease risk, and dementia-related blood biomarker levels in a population-based cohort study followed over 17 years

Appendices Contents:

1. **Supplementary Text 1**……………………………..……………………………………………………………………..2
2. **Supplementary Text 2**…………………………………………………………………………………………………….3
3. **Supplementary Figure 1**. Flow chart of ESTHER participants included in the study…….…..4
4. **Supplementary Figure 2**. Distribution of the polygenic risk scores (Kunkle et al. 2019 and Bellenguez et al. 2022) in the ESTHER study 5
5. **Supplementary Table 1**. SNPs included in the polygenic risk scores………………………..……...6
6. **Supplementary Figure 3**. Correlation between the polygenic risk scores and AD related blood biomarkers 8
7. **Supplementary Table 2**. Participant characteristics and association to blood-based biomarkers, P-tau181, NfL, & GFAP 9
8. **Supplementary Table 3.** Age at diagnosis according to APOE4 status and PRS quartile...10
9. **Supplementary Table 4**. Association of *APOE* and PRSs with Alzheimer’s disease, All-cause dementia, and blood biomarkers stratified by sex …………………………………....………11

**Supplementary Text 1**:

ESTHER participants completed standardized health questionnaires, provided blood and urine samples, and GPs provided medical information. Comprehensive monitoring of major disease incidence and mortality was conducted through participant and GP follow-up 2, 5, 8, 11, 14, and 17 years after recruitment for all participants. Furthermore, data were linked to the Saarland Cancer Registry and death certificates were obtained from local health authorities.

GPs were asked to fill out questionnaires regarding the participants’ dementia status (i.e. presence of dementia diagnosis, type of dementia, dementia diagnosis date, or confirmed lack of dementia diagnosis) and provide all available medical records from specialists such as neurologists or psychiatrists. All-cause dementia diagnoses include unspecified or unknown dementia. The current guidelines in Germany for AD diagnosis follow the National Institute on Aging and the Alzheimer’s Association^1^, ICD-10, or the International Working group (IWG)-2 criteria, for VD diagnosis the National Institute of Neurological Disorders and Stroke (NINDS)- Association Internationale pour la Recherche et l'Enseignement en Neurosciences (AIREN) criteria,^2^ and all-cause dementia diagnoses are recommended if the dementia symptoms outlined by the ICD-10 are present for at least six months.^3^

**Supplementary Text 2**:

DNA from whole blood samples was collected using a salting out procedure. The extracted DNA from blood cells was genotyped using the Illumina Infinium OncoArray and Global Screening Array BeadChips (Illumina, San Diego, CA, USA). General genotyping quality control assessment was done following the Nature Protocols article from Anderson et al.^29^ Imputation of the quality controlled data was conducted using the Michigan Imputation Server, where SHAPEIT2 was used to phase the data, and MiniMac 4 was used to impute to the HRC Version r1.1 2016 reference panel.^30,31^ *APOE* genotyping was performed using Taqman single nucleotide polymorphism (SNP) assays and in the case of missing directly genotyped data, available quality controlled, imputed genetic data was utilized (5% of *APOE* data). Simoa technology was used to measure P-tau181, GFAP, and NfL in plasma drawn at baseline in a single batch.^27^ Briefly, lithium-Heparin samples were measured utilizing the commercially available Simoa Neurology 4-Plex E Advantage Kit and Simoa pTau-181 Advantage V2 Kit (Quanterix, MA, USA) on the Simoa HD-X Analyzer according to manufacturer´s instructions at the Ruhr-University Bochum, Bochum, Germany. All laboratory measurements were performed in a blinded fashion. Serum creatinine measurements were performed by the kinetic Jaffe method and serum cystatin C concentrations were measured by immunonephelometry on a Behring Nephelometer II (Dade-Behring Diagnostic, Marburg, Germany).

**Supplementary Figure 1. Flow chart of ESTHER participants included in the study.** Incident dementia cases included participants that received a dementia diagnosis between baseline and the 17-year follow-up. Controls included participants that remained without dementia diagnosis (GP confirmed) throughout follow-up.

*P-tau181, GFAP, and NfL levels in blood plasma

**n = 105:** participants with GPs that could not be contacted due to misc. reasons (e.g. address changes)

Dementia diagnosis information from GP requested 2016-2020, n **= 8353**

ESTHER Baseline 2000-2002.

**n = 9940**

Dementia diagnosis information from

GP received 2016-2020, n **= 6528**

Total number of participants with usable dementia information from GP at 17-year follow-up, **n=6357**

**n = 248:** participant consent to contact GP withdrawn

**n = 930:** participants with GPs that could not be contacted due to closure of practice, retirement, or death

**n = 1413**: participants with no GP response

**n = 412**: participants with GPs without available dementia diagnosis information

**n = 171**: participants with suspected but not confirmed dementia diagnosis

**n = 304:** participants with GP consent to be contacted withdrawn

Incident dementia cases

**n = 464**

Controls

**n = 5301**

**n = 592**: participants without available genetic information

Nested case-control sample:

**Baseline** AD-related plasma biomarker measurements*, n **= 768**

Incident dementia cases

**n = 239**

Controls

**n = 470**

**n =53**: participants without available genetic information

**n =3**: participants with measurements that failed quality control


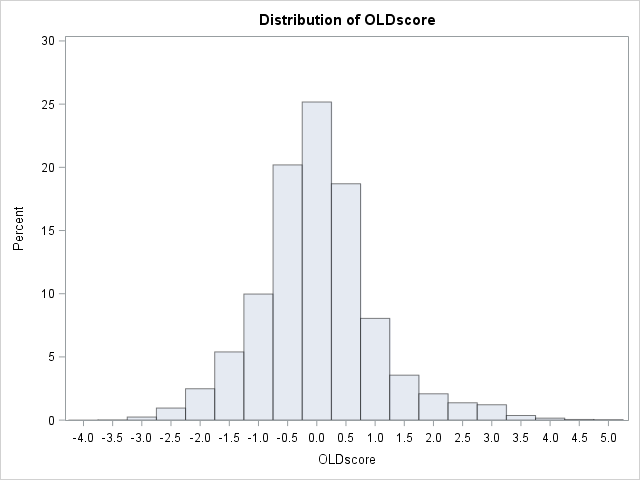

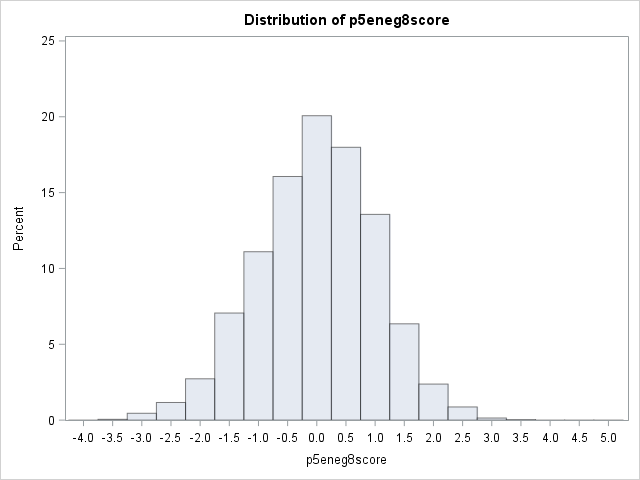


Kunkle score

Bellenguez score

**Supplementary Figure 2.** **Distribution of the polygenic risk scores (Kunkle et al. 2019 and Bellenguez et al. 2022) in the ESTHER study**

| **Supplementary Table 1**. SNPs included in the polygenic risk scores | | | | | |
| --- | --- | --- | --- | --- | --- |
| Bellenguez SNPs (rsnames) | Kunkle SNPs (rsnames) | | | |  |
| \| **rs13032148** \| rs5026743 \| rs11257240 \| **rs13032148** \| rs652354 \| \| --- \| --- \| --- \| --- \| --- \| \| **rs35103166** \| rs2760994 \| rs10769256 \| **rs35103166** \| rs11218343 \| \| **rs11762262** \| rs6926479 \| rs10838709 \| **rs11762262** \| rs3764650 \| \| **rs580064** \| rs35305377 \| rs632185 \| **rs580064** \| rs111358663 \| \| **rs611267** \| rs1065712 \| rs671686 \| **rs611267** \| rs2965101 \| \| **rs3844143** \| rs35505579 \| rs666682 \| **rs3844143** \| rs2927437 \| \| **rs3795065** \| rs751141 \| rs646260 \| **rs3795065** \| rs35106910 \| \| **rs7255066** \| rs2011567 \| rs72962020 \| **rs7255066** \| rs148933445 \| \| **rs62117162** \| rs13276936 \| rs60228070 \| **rs62117162** \| rs147711004 \| \| **rs3208856** \| rs7068231 \| rs7127056 \| **rs3208856** \| rs34278513 \| \| **rs140824606** \| rs583296 \| rs8019585 \| **rs140824606** \| rs11668861 \| \| **rs111371860** \| rs11230235 \| rs11854073 \| **rs111371860** \| rs73052307 \| \| **rs1871047** \| rs4354705 \| rs2555356 \| **rs1871047** \| rs73936968 \| \| **rs365653** \| rs10897048 \| rs9972866 \| **rs365653** \| rs118170342 \| \| **rs79701229** \| rs598561 \| rs62054657 \| **rs79701229** \| rs405509 \| \| **rs283813** \| rs683494 \| rs62063276 \| **rs283813** \| rs12691088 \| \| **rs1160984** \| rs631611 \| rs7502672 \| **rs1160984** \| rs73033507 \| \| **rs1064725** \| rs7519119 \| rs72973584 \| **rs1064725** \| rs1114832 \| \| **rs144311893** \| rs114644769 \| rs80257887 \| **rs144311893** \| rs12460985 \| \| **rs12721109** \| rs35589443 \| rs112027066 \| **rs12721109** \| rs56187099 \| \| **rs1132899** \| rs76168490 \| rs62120565 \| **rs1132899** \|  \| \| **rs35194062** \| rs6743470 \| rs2927488 \| **rs35194062** \|  \| \| **rs874743** \| rs11680911 \| rs534178240 \| **rs874743** \|  \| \| **rs112481437** \| rs28534487 \| rs1466435 \| **rs112481437** \|  \| \| **rs2627641** \| rs2544659 \| rs41290100 \| **rs2627641** \|  \| \| **rs189063316** \| rs28732234 \| rs393584 \| **rs189063316** \|  \| \| rs72843896 \| rs9270599 \| rs41290108 \| rs11118328 \|  \| \| rs11694743 \| rs9275207 \| rs11667640 \| rs10929006 \|  \| \| rs11554586 \| rs3997854 \| rs769448 \| rs3135348 \|  \| \| rs62375411 \| rs4727449 \| rs204468 \| rs114812713 \|  \| \| rs9378202 \| rs1727128 \| rs34827707 \| rs755951 \|  \| \| rs28732216 \| rs5015755 \| rs2041262 \| rs2279590 \|  \| \| rs12546365 \| rs10228407 \| rs111243475 \| rs3740688 \|  \| \| rs11257101 \| rs7341557 \| rs346764 \| rs12798346 \|  \| \| rs1788283 \| rs4732728 \|  \| rs4752801 \|  \| \| rs469420 \|  \|  \|  \|  \| \| Note: Overlapping SNPs are in red and bolded. \| \| \| \| \| | |  |  |  |  |
|  | |  |  |  |  |


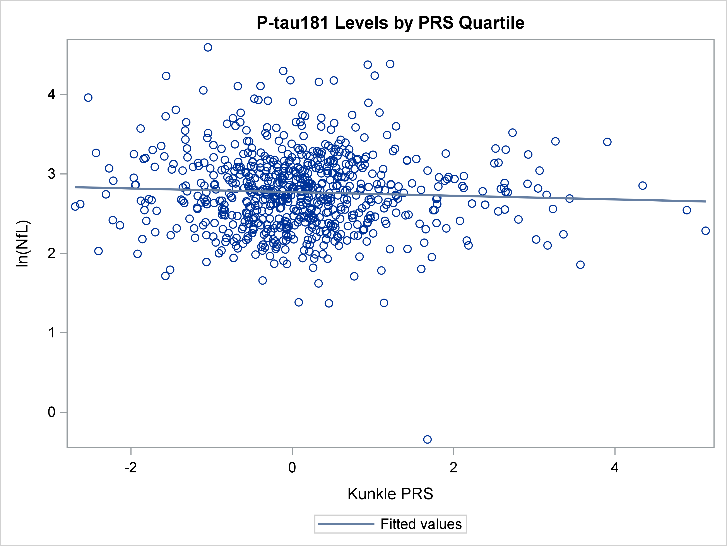

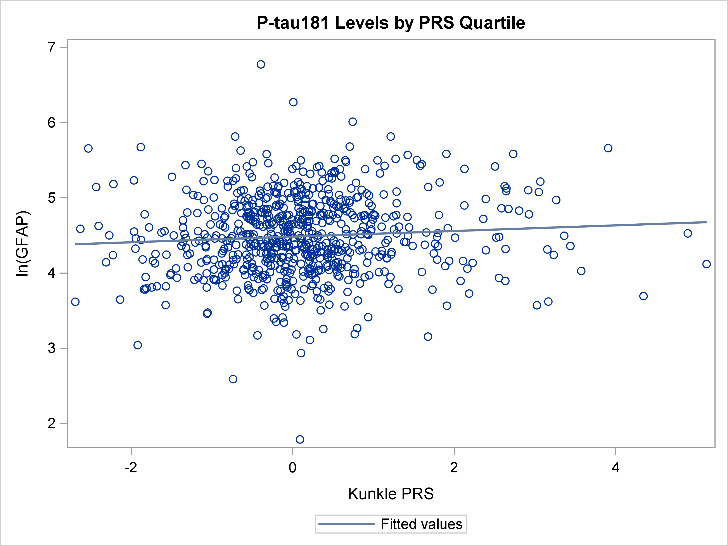

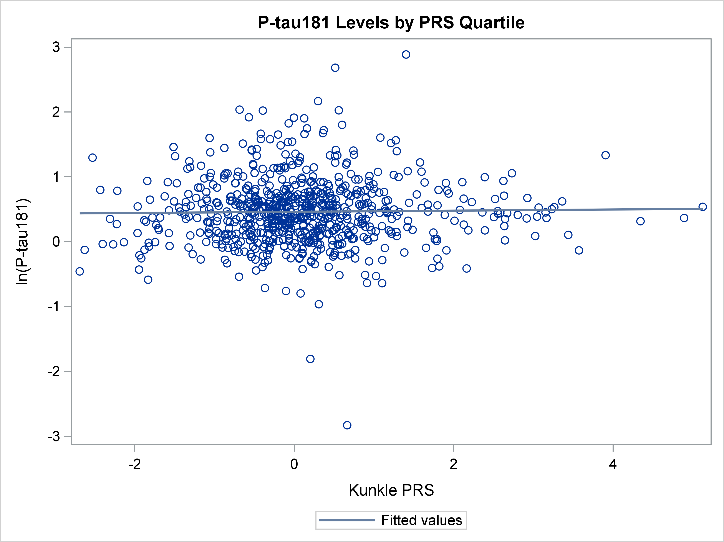


r=0.02, p=.63


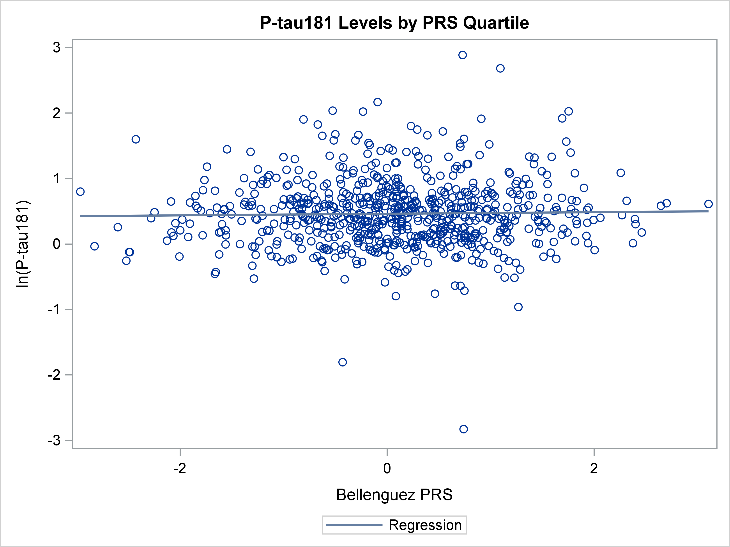

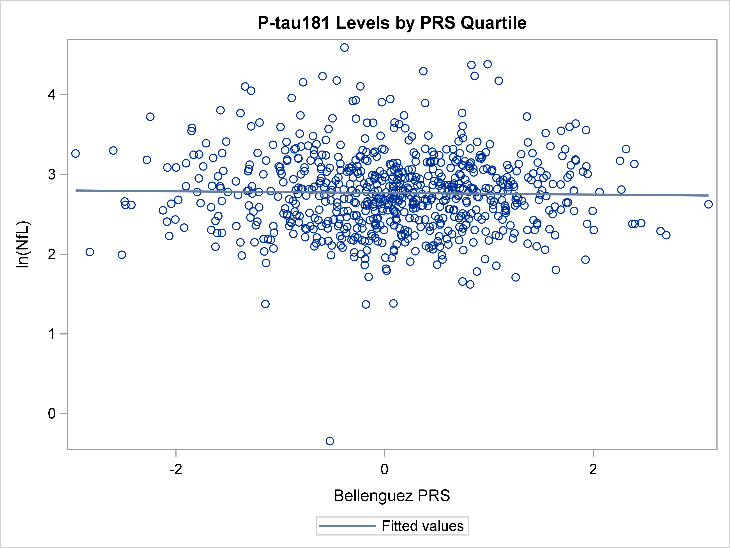


r=-0.02, p=.59


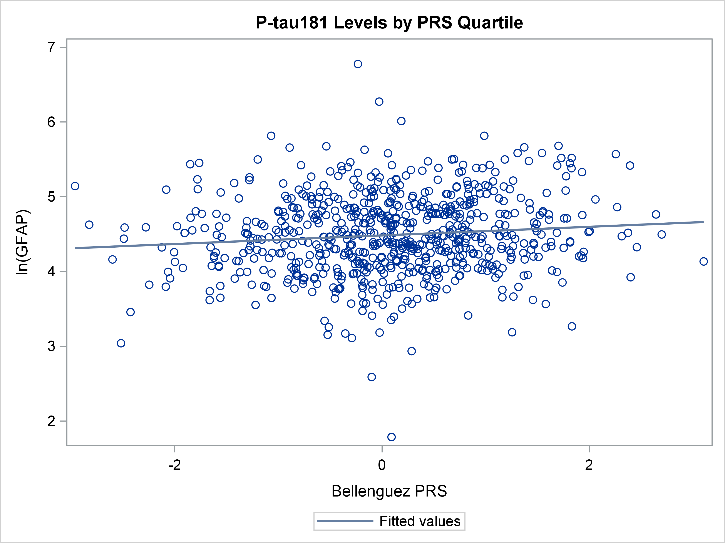


r=0.02, p=.54

r=-0.05, p=.19

r=0.07, p<.05

r=0.10, p<.01

**Supplementary Figure 3.** **Correlation between the polygenic risk scores and AD related blood biomarkers**. The Pearson correlation coefficients (r) between biomarker and PRS values as well as the regression line are shown for each log-transformed biomarker: P-tau181 [A, D], NfL [B, E], and GFAP [C, F]; Kunkle PRS, [A, B, C]; Bellenguez PRS [D, E, F].

**A**

**B**

**C**

**D**

**E**

**F**

| Supplementary Table 2. Participant characteristics and association to blood-based biomarkers, P-tau181, NfL, & GFAP | | | | | | | | |  |
| --- | --- | --- | --- | --- | --- | --- | --- | --- | --- |
|  | *P-tau181* | | *NfL* | | *GFAP* | | P-tau181 | *NfL* | *GFAP* |
| Predictor | *Top Quintile*  n (%) | *Lower Quintiles*  n (%) | *Top Quintile*  n (%) | *Lower Quintiles*  n (%) | *Top Quintile*  n (%) | *Lower Quintiles*  n (%) | *Top Quintile vs. Lower Quintiles*  OR (95%CI) | *Top Quintile vs. Lower Quintiles*  OR (95%CI) | *Top Quintile vs. Lower Quintiles*  OR (95%CI) |
| n | 142 | 570 | 142 | 567 | 141 | 568 |  |  |  |
| Age, mean ± SD  (Range) | 66.5 ± 6.2  (51-75) | 62.4 ± 6.6  (50-75) | 69.0 ± 4.6  (52-75) | 61.8 ± 6.4  (50-75) | 68.1 ± 5.4  (51-75) | 62.0 ± 6.5  (50-75) | **1.09 (1.05-1.12) <.0001** | **1.23 (1.17-1.28) <.0001** | **1.18 (1.13-1.23) <.0001** |
| Male  Female | 55 (38.7)  87 (61.6) | 307 (53.9)  263 (46.1) | 75 (52.8)  67 (47.2) | 240 (42.3)  327 (57.7) | 47 (33.3)  94 (66.7) | 300 (52.8)  268 (47.2) | Ref.  1.35 (0.91-2.00) .16 | Ref.  **0.51 (0.33-0.80) .003** | Ref.  **0.49 (0.33-0.75) .0009** |
| *APOE4* –  *APOE4* + | 84 (59.6)  57 (40.4) | 402 (70.7)  167 (29.4) | 94 (66.2)  48 (33.8) | 389 (68.9)  176 (31.1) | 79 (56.0)  62 (44.0) | 404 (71.4)  162 (28.6) | Ref.  **1.60 (1.07-2.39) .02** | Ref.  1.14 (0.72-1.81) .57 | Ref.  **1.95 (1.28-2.97) .002** |
| Kunkle PRS  per SD increase | - | - | - | **-** | **-** | **-** | 0.90 (0.74-1.10) .26 | 0.95 (0.77-1.18) .64 | **1.23 (1.01-1.48) .04** |
| Bellenguez PRS  per SD increase | - | - | - | **-** | **-** | **-** | 0.94 (0.77-1.15) .52 | 0.80 (0.63-1.00) .05 | 1.19 (0.96-1.47) .12 |
| Note: Logistic regression analyses adjusted for age, sex, 10 principal components, and estimated glomerular filtration rate according to the 2021CKD-EPI creatinine-cystatin C equation (eGFRcr-cys). Bold values denote statistical significance at the p < .05 level. Abbreviations: *APOE*4 +, 1 or more e4 alleles, *APOE*4 -, no e4 alleles, CI, confidence interval, OR, odds ratio, SD, standard deviation, Q, quartile | | | | | | | | | |

| **Supplementary Table 3. Age at diagnosis according to *APOE4* status and PRS quartile** | | | | | | |
| --- | --- | --- | --- | --- | --- | --- |
| Predictor | n | Alzheimer’s disease  Age at diagnosis, mean ± SD | p-value* | n | All-cause dementia  Age at diagnosis, mean ± SD | p-value* |
| *APOE4*-  *APOE4*+ | 76  76 | 77.7 ± 6.0  76.4 ± 4.8 | .15 | 181  280 | 78.1 ± 5.9  77.4 ± 5.1 | .18 |
| Kunkle et al. score  Q1  Q2  Q3  Q4 | 27  32  35  59 | 79.4 ± 5.8  75.7 ± 5.9  78.0 ± 5.1  76.1 ± 4.9 | .**02** | 103  102  103  156 | 78.0 ± 5.7  77.1 ± 5.8  79.0 ± 5.2  77.3 ± 5.6 | .46 |
| Bellenguez et al. score  Q1  Q2  Q3  Q4 | 24  39  40  50 | 79.2 ± 5.4  77.5 ± 5.9  76.2 ± 5.3  76.3 ± 5.1 | .13 | 98  105  122  139 | 78.3 ± 5.4  77.7 ± 5.9  78.1 ± 5.2  77.2 ± 5.8 | .07 |
| **Note**: *p-values for difference in age at diagnosis by genetic risk predictor according to t-test or ANOVA analysis. | | | | | | |

| **Supplementary Table 4**. Association of *APOE* and PRSs with Alzheimer’s disease, All-cause dementia, and blood biomarkers stratified by sex | | | | | | | | |
| --- | --- | --- | --- | --- | --- | --- | --- | --- |
|  | Men | | |  | Women | | |  |
|  | Total  n | Cases  n | OR (95% CI) p-value |  | Total  n | Cases  n | OR (95% CI) p-value | Interaction  p-value |
| *Alzheimer’s disease* |  |  |  |  |  |  |  |  |
| *APOE*4 –  *APOE*4 + | 2474 | 67 | Ref.  **2.54 (1.54-4.18) .0002** |  | 2947 | 85 | Ref.  **4.13 (2.63-6.49) <.0001** | .21 |
| Kunkle PRS  per SD increase | 2490 | 67 | **1.29 (1.02-1.64) .03** |  | 2964 | 86 | **1.69 (1.38-2.06) <.0001** | .10 |
| Bellenguez PRS  per SD increase | 2490 | 67 | 1.26 (0.98-1.63) .07 |  | 2964 | 86 | **1.36 (1.09-1.70) .007** | .81 |
| *All-cause dementia* | | | |  |  |  |  |  |
| *APOE*4 –  *APOE*4 + | 2634 | 227 | Ref.  **2.09 (1.56-2.79) <.0001** |  | 3096 | 234 | Ref.  **2.24 (1.66-3.01) <.0001** | .94 |
| Kunkle PRS  per SD increase | 2650 | 227 | 1.11 (0.97-1.29) .12 |  | 3115 | 237 | **1.35 (1.18-1.55) <.0001** | .08 |
| Bellenguez PRS  per SD increase | 2650 | 227 | 1.13 (0.98-1.30) .09 |  | 3115 | 237 | **1.15 (1.00-1.33) .048** | .99 |
| *P-tau181 beta (p-value) beta (p-value)* | | | | | | | | |
| *APOE*4 | 317 |  | 0.08 (.16) |  | 393 |  | 0.06 (.28) | .41 |
| Kunkle PRS  per SD increase | 318 |  | .009 (.70) |  | 394 |  | 0.007 (.79) | .63 |
| Bellenguez PRS  per SD increase | 318 |  | 0.006 (.81) |  | 394 |  | 0.003 (.91) | .77 |
| *NfL* | | | | | | | | |
| *APOE*4 | 314 |  | -0.04 (.43) |  | 393 |  | -0.05 (.23) | .71 |
| Kunkle PRS  per SD increase | 315 |  | -0.02 (.44) |  | 394 |  | -0.02 (.23) | .46 |
| Bellenguez PRS  per SD increase | 315 |  | -0.02 (.41) |  | 394 |  | -0.001 (.95) | .84 |
| *GFAP* | | | | | | | | |
| *APOE*4 | 314 |  | **0.13 (.02)** |  | 394 |  | 0.03 (.59) | .14 |
| Kunkle PRS  per SD increase | 315 |  | **0.05 (.06)** |  | 394 |  | 0.02 (.36) | .26 |
| Bellenguez PRS  per SD increase | 315 |  | **0.08 (.005)** |  | 394 |  | 0.03 (.22) | .04 |
| Note: Logistic regression analyses adjusted for age, sex, and 10 principal components. The analyses with the blood biomarkers as outcomes additionally adjusted for the estimated glomerular filtration rate according to the 2021CKD-EPI creatinine-cystatin C equation (eGFRcr-cys). Bold values denote statistical significance at the p < .05 level.  Abbreviations: *APOE*4 +, 1 or more e4 alleles, *APOE*4 -, no e4 alleles, CI, confidence interval, OR, odds ratio, SD, standard deviation, Q, quintile | | | | | | | | |
